# Toxicity and Sublethal Effect of Chlorantraniliprole on Multiple Generations of *Aedes aegypti* L. (Diptera: Culicidae)

**DOI:** 10.3390/insects15110851

**Published:** 2024-10-30

**Authors:** Nimra Batool, Muhammad Abubakar, Ahmed Noureldeen, Muhammad Nadir Naqqash, Akram Alghamdi, Zamzam M. Al Dhafar, Fadi Baakdah, Raimondas Mozūratis

**Affiliations:** 1 Institute of Plant Protection, MNS University of Agriculture, Multan 59220, Pakistan; nimrabatool1133@gmail.com; 2 Department of Entomology, Bahauddin Zakariya University, Multan 60800, Pakistan; abubakar.m3962@gmail.com; 3 Department of Biology, College of Sciences, Taif University, Taif 21944, Saudi Arabia; a.noureldeen@tu.edu.sa (A.N.); a.alghamdii@tu.edu.sa (A.A.); 4 Department of Biology, College of Science, Imam Abdulrahman Bin Faisal University, P.O. Box 1982, Dammam 31441, Saudi Arabia; zaldhafar@iau.edu.sa; 5 Basic and Applied Scientific Research Center (BASRC), Imam Abdulrahman Bin Faisal University, P.O. Box 1982, Dammam 31441, Saudi Arabia; 6 Department of Medical Laboratory Sciences, Faculty of Applied Medical Sciences, King Abdulaziz University, Jeddah 21589, Saudi Arabia; fbaakdah@kau.edu.sa; 7 Special Infectious Agents Unit, King Fahd Medical Research Center, King Abdulaziz University, Jeddah 21589, Saudi Arabia; 8 Laboratory of Chemical and Behavioral Ecology, Institute of Ecology, Nature Research Centre, LT-08412 Vilnius, Lithuania; 9 Department of Zoology, Stockholm University, SE-10691 Stockholm, Sweden

**Keywords:** life table, transgenerational studies, metabolic enzymes, hermetic effect, TWOSEX-MSChart

## Abstract

Due to the quick development of insecticide resistance, it is crucial to optimize management programs by understanding the sublethal effects of effective insecticides like chlorantraniliprole on *Aedes aegypti* L. populations. Using age-stage and two-sex life tables, we investigated the sublethal impacts of chlorantraniliprole on *Ae. aegypti*. Larval duration in the progeny of exposed parents was reduced by 0.33–0.42 days, whereas, the longevity of male and female adults was decreased by 1.43–3.05 days. Similarly, the egg-laying capacity of F_1_ and F_2_ progeny of the exposed parents was significantly reduced from 27.3% to 41.2%. The mean generation time (*T*) increased up to 11.8% in exposed populations, and the net reproduction rate (*Ro*) decreased by 51.50–55.60%. After 24 h of chlorantraniliprole treatment, there was a significant increase in cytochrome P450 activity. Contrarily, the activity of glutathione S-transferase (GST) initially declined but started increasing after 48 h of treatment. This research highlights the importance of chlorantraniliprole in mosquito management, as well as the importance of considering sublethal effects when developing strategies to handle them. Having a thorough understanding of the harmful effects of insecticides on mosquito populations can greatly enhance the effectiveness of insecticide-based interventions, while also minimizing the risk of pest resurgence.

## Simple Summary

Mosquitoes are vectors of various diseases in humans. Due to the quick development of insecticide resistance, it is crucial to optimize management programs by understanding the sublethal effects of effective insecticides like chlorantraniliprole on *Aedes aegypti* L. populations. For this purpose, a population of *Ae. aegypti* was reared in the laboratory for 15 generations then exposed to chlorantraniliprole and sublethal effects were studied on F_1_ and F_2_ generations. The following life history parameters were decreased significantly in exposed F_1_ and F_2_ generations compared to the control: larval duration, male longevity, female longevity, and oviposition days. The adult preoviposition and total preoviposition period did not differ significantly. The fecundity of *Ae. aegypti* in F_1_ generation decreased from 61 eggs/female in the control to 34 eggs/female in LC_50_. In conclusion, our findings demonstrate that sublethal doses of chlorantraniliprole have significant transgenerational effects on *Ae. aegypti* mosquitoes. The cautious usage of chlorantraniliprole is recommended for the effective management of mosquitoes and to lessen long-term influence on human health and the environment.

## 1. Introduction

The incidence of developing and reemerging mosquito-borne diseases has posed severe worldwide public health issues [1]. Outbreaks of mosquito-borne sicknesses, which include dengue, chikungunya, and Zika, have been an extreme burden on the economies, healthcare systems, and populations of the affected countries. Many cases have been documented in several Asian countries, including Indonesia, Vietnam, the Philippines, Bangladesh, and Malaysia. In October 2018, France and Spain reported the first dengue fever cases from within the country [2]. The fact that dengue fever has lately appeared in the United States and Japan suggests that it is no longer limited to tropical areas [3,4].

The effectiveness of mosquito control is hindered by a narrow range of insecticides and growing resistance to these insecticides [5], This has clear implications for the occurrence of diseases transmitted by *Aedes aegypti* L. (Diptera: Culicidae), such as dengue, yellow fever, chikungunya, and Zika fever [6]. Due to the quicker development of insecticide resistance, it is critical to assess insecticide use thoroughly. Although widespread insecticide usage in agriculture continues to fuel the rise of insecticide resistance, integrated pest management (IPM) programs stress the need for moderate pesticide use [7].

Chlorantraniliprole is considered one of the most effective insecticides in the group of ryanodine receptor modulators. It can effectively control lepidopteran pests, including those that have developed resistance to other pesticides [8]. Furthermore, there is a significant preference for ryanodine receptors present in insects as opposed to those present in vertebrates [9]. When ingested, chlorantraniliprole changes the activity of the ryanodine receptor, therefore causing calcium release from inside cells. The insect dies due to having stopped feeding, become sluggish, and developed paralysis in some muscles [10]. Furthermore, chlorantraniliprole most efficiently targets lepidopteran and some coleopteran and dipteran insects [11] while showing less toxicity to bees [12] as well as to most predatory and parasitic insects [13,14]. Because anthranilic diamides, including chlorantraniliprole, do not harm vertebrates [9] and have lower toxicity to many beneficial insects, these insecticides are good choices for controlling mosquitoes and other insect pests [15,16].

Sublethal doses are insecticide doses under the median lethal dose, i.e., LC_50_. These are not administered to gather mortality data; instead, their chronic effects are studied, which are characterized by deleterious physiological and/or behavioral changes leading to reduced fitness [17]. Sublethal behavioral and physiological changes brought on by insecticides may impact population dynamics. For instance, chemicals can reduce an insect’s life span and fecundity [17,18]. In contrast, research on other insect pests, including *Frankliniella occidentalis* Pergande (Thysanoptera: Thripidae) [19], *Rhopalosiphum padi* L. (Hemiptera: Aphididae) [20], and *Myzus persicae* [21], has shown that sublethal effects of insecticide exposure can enhance biotic potential. The phenomenon of insecticides causing a stimulatory effect is referred to as “insecticide-induced hormesis”. Hormesis is a phenomenon in which a small amount of a substance stimulates a response, whereas a big dose inhibits it. This response is a moderate overcompensation to a disturbance in the body’s balance or a direct stimulatory effect [22,23,24]. Understanding the overall effects on pests and the sublethal effects on non-targeted arthropods is thus of the utmost importance.

The lethal effects of pyrethroid-type insecticides on mosquitoes have been recently reviewed [25]; however, to our knowledge, the sublethal or hermetic effects of chlorantraniliprole in *Ae. aegypti* have not been examined. This study aims to obtain a comprehensive picture of the sublethal effects of chlorantraniliprole on different biological traits and demographic aspects of *Ae. aegypti* using the age-stage and two-sex life table. To maximize the use of this insecticide in control programs for *Ae. aegypti* and hence increase its efficacy, one must understand its hermetic or deleterious effect on subsequent generations.

## 2. Materials and Methods

### 2.1. Insect Rearing

Mosquitoes were collected from stagnant water in areas with low insecticide levels in the vicinity of Riyadh, Saudi Arabia, in areas where no or few insecticides were used, to minimize the effect of other factors before conducting this study according to the predefined methodology of an earlier study conducted by Rahman et al. [7]. Approximately 200 larvae from each strain were individually placed in plastic basins containing one liter of distilled water. Fish meal (TetraMin^®^) was consistently fed to the larvae every 5 days to satisfy their nutritional demands till pupation. A dosage of one gram was administered to each plastic basin. The pupae were carefully moved to wooden mesh adult cages (30 × 30 × 30 cm) for adult emergence. Adult *Ae. aegypti* were provided with a 10% (*w*/*v*) sugar solution. To provide female *Ae. aegypti* mosquitoes with blood, a pigeon (*Columba livia* L.) was placed in each cage three times weekly. To further aid in the depositing of eggs, each cage had filter paper placed in a glass beaker half-filled with water [7]. Mosquitoes were reared for more than 15 generations before starting the transgenerational experiments.

### 2.2. Insecticides

Coragen^®^, a commercially available formulation of chlorantraniliprole that is a 20% SC (suspension concentrates) imported by FMC Pvt. Ltd., Lahore, Pakistan was used in the study.

### 2.3. Mortality Bioassays

A larval bioassay was conducted to evaluate the toxicity of chlorantraniliprole [26]. A 600 mL stock solution of pesticide was made using distilled water. Seven concentrations were prepared from the stock solution, ranging from 0.15 to 4.8 mg/L (including one control). The concentrations selected were based on achieving a mortality range b (Version 1.0) etween 0% and 99% according to previously defined methodology. Three replicates were performed for each concentration, with each replicate consisting of 20 larvae. A total of 420 larvae were used in a bioassay, including the control. The cups were stored in a controlled laboratory environment at a temperature of 28 ± 2 °C and a relative humidity of 65 ± 5%. The death rate was evaluated 72 h after exposure.

### 2.4. Sublethal Effects on Demographic Features

The effect of a sublethal dose on the development of *Ae. aegypti* was evaluated after 15 generations, i.e., the 16th generation was labelled as F_0_ and used as the parent generation for bioassays (without selection pressure). Two sublethal concentrations (LC_10_ and LC_30_), one lethal concentration (LC_50_), and a control were used to study the effects on the F_0_, F_1_, and F_2_ generations. The modified approach of Shafi et al. [27] was employed for this purpose. Each concentration was used to inoculate 50 third-instar larvae. After a duration of 72 h, the individuals that remained alive from each of the three doses were cleaned and placed in a fresh larval container containing 1000 mL of tap water with larval feed (0.5 g every 2 days). After pupation, the pupae were transferred to a plastic container with 100 mL of tap water and then moved to a new adult cage for the purpose of hatching. After 5–6 days of adult emergence, the females were provided with a blood meal to facilitate egg laying. The eggs were collected from these mature individuals and then moved to a larval box to facilitate hatching. The oviposition of test female mosquitoes was individually induced in glass Petri dishes (6 cm in diameter) lined with moist filter paper.

The biological properties of all strains of *Ae. aegypti* (F_1_ and F_2_ reared at LC_10_, LC_30_, LC_50_, and the control) were individually investigated following Maimusa et al. [28]. Factors such as fecundity, fertility (the rate at which females lay eggs), and adult survival rate were considered. Fifty newly hatched first-instar larvae from each strain were placed inside a plastic basin filled with 500 mL of distilled water. The previously mentioned method was used to care for and raise the larvae. To facilitate the emergence of the adult insects, the pupae were moved to adult cages. Following the hatching of the adult mosquitoes from their larval stage, the researchers meticulously recorded the proportion of males to females. Mosquito adults were exposed to a 10% (*w*/*v*) sugar–water solution. The survival rate of several mosquito life cycle stages was monitored daily using assessments. The number of fertile females was recorded after 24 h. The quantity of eggs produced by each female was documented till the death of all individuals. The conditions for adult rearing are already mentioned above.

### 2.5. P450 and GST Activity

The approach employed by Habig et al. [29] was utilized to assess GST activity, utilizing 1 mmol/L 1-chloro-2,4-dinitrobenzene (CDNB) as the substrate for the reaction. Absorbance variations were observed over a duration of 4 min, recorded at 30 s intervals, specifically at a wavelength of 340 nm. The activity of P450 monooxygenase was assessed using the substrate *p*-nitroanisol in conjunction with the NADPH system, as described by Kristensen et al. [30]. The NADPH generating system comprised 12 mL of 100 mM phosphate buffer (pH 7.5), 250 µL of 10 mM NADP, 250 µL of 100 mM glucose-6-phosphate, and 12 µL of 100 U glucose-6-phosphate dehydrogenase. An amount of 250 µL of PNA was incorporated into the NADPH generating system for the mixture. Each well comprised 100 µL of sample and 100 µL of the final cocktail. The reaction rate was assessed by monitoring the formation of PNA at 405 nm at a temperature of 37 °C, utilizing a kinetic program over 20 min, with measurements taken every 2 min and an initial lag time of 10 min.

### 2.6. Statistical Analysis

Values for the 95% fiducial limit (FLs), chi-squared (X^2^), degree of freedom (df), and slope with standard error of chlorantraniliprole bioassays in *Ae. aegypti* were calculated for LC_10_, LC_30_, and LC_50_ by probit analysis using PoloPlus software (Version 1.0) [31]. The data were analyzed using the age-stage and two-sex life table hypothesis to create a life table. The population characteristics investigated in this work comprised the finite rate of rise (*λ*), the mean generation time (*T*), the net reproduction rate (*R*_0_), and the intrinsic rate of increase (*r*) [32,33,34,35].

The value of *l_x_m_x_* was calculated using the equation

$$\sum_{x=0}^{\infty }e^{-r(x+1)}lxmx=1$$

Age-stage specific survival can be calculated using the statistical formula

$$lx=\sum_{j=1}^{k}Sxj$$

*R_o_* was calculated using the equation

$$R_{0}=\sum_{x=0}^{\infty }l_{x}m_{x}$$

The equation to determine the *T* value was

*T* = (In *Ro*)/*r*

The value of *r* was estimated by

$$\sum_{x=0}^{\infty }e^{-r(x+1)}lxmx=1$$

The finite rate of increase was calculated by

*Λ* = *e*^*r*^

The value *e_xj_* was determined by

$$e_{\mathrm{xj}}=\sum_{i=x}^{\infty }\sum_{j=y}^{k}S_{\mathrm{ij}}$$

The *V_xj_* value was determined with equation

$$V_{xj}=\frac{e^{-r(x+1)}}{S_{xj}}\sum_{i=x}^{\infty }e^{-r(i+1)}\sum_{j=y}^{k}S_{ij}f_{ij}$$

The following life table parameters were measured: age-specific survival rate (*l_x_*); female age-specific fecundity (*fx*); age-specific fecundity (*m_x_*); mean fecundity of individuals at age *x*; and age-specific maternity (*l_x_m_x_*). The demographic variables, including *R*_0_, *r*, *λ*, and *T*, were determined using the TWOSEX-MSChart tool. We used the bootstrap procedure with a sample size of 100,000 to estimate the means, standard errors (SE), and variances. This approach yielded reduced variability in the results and a frequency distribution that followed a normal pattern. Furthermore, the variation in sample sizes did not impact the conclusions. Results with significant differences were compared by paired bootstrap testing [36]. The graphs were generated exclusively with SigmaPlot 12.0 (Systat Software Inc., San Jose, CA, USA).

## 3. Results

### 3.1. Toxicity of Chlorantraniliprole on Ae. aegypti Larvae

The LC_50_ value of chlorantraniliprole in the parental population of *Ae. aegypti* was 0.569 mg L^−1^, with 95% FLs of 0.483–0.663 mg L^−1^. The LC_10_ and LC_30_ values with their 95% FLs were 0.202 (0.145–0.257 mg L^−1^) and 0.373 (0.300–0.443 mg L^−1^), respectively, (Table 1). All three lethal concentration (LC) values were significantly different from each other, as their 95% FL values did not overlap. Sublethal tests were conducted using these values.

### 3.2. Sublethal Effects of Chlorantraniliprole on the Parental (F_0_) Generation

**Figure 1:**
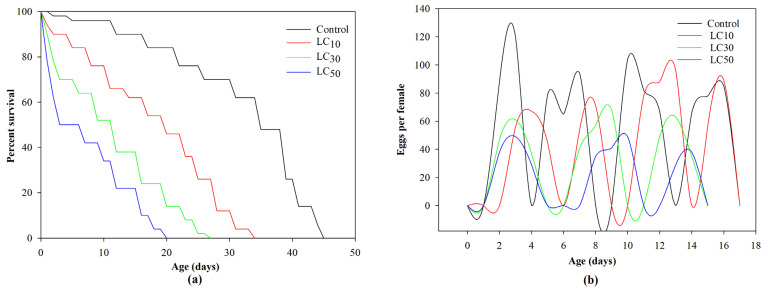
Effect of different concentrations (LC_10_, LC_30_, LC_50_, and control) of chlorantraniliprole on survival rate (**a**) and fecundity (**b**) of parental generation (F_0_) of *Ae. aegypti*.

The control population had a higher survival rate than insecticide-treated *Ae. aegypti* populations. Control insects survived up to 45 days. In contrast, the survival rate in LC_50_ treatment fell to zero on the 27th day. Similarly, the control group had the most eggs (928), followed by LC_10_, LC_30_, and LC_50_ (Figure 1).

### 3.3. Transgenerational Effects of Chlorantraniliprole on Different Biological Parameters

A modest concentration of chlorantraniliprole had a significant effect on the time it took for the F_1_ generation of *Ae. aegypti* to grow and breed (Table 2). There were no significant variations in the time it took for eggs to hatch in either the F_1_ or F_2_ offspring of the populations. The control progenies showed significantly longer larval durations, ranging from 6.55 to 6.61 days. The F_1_ and F_2_ progenies of the LC_50_ treatment had significantly lower larval durations, ranging from 6.21 to 6.25 days. There were no significant differences in pupal lengths, APOP, or TPOP across the treatments’ progenies. Both male and female adults lived longer in the control offspring (F_1_ and F_2_) than the progenies treated. The control females produced significantly more eggs (59.34–61.08 eggs/female) than the females from the other treatments. Female insects subjected to the LC_50_ treatment deposited significantly fewer eggs, ranging from 34.78 to 38.55 per female, compared to the rest of the treatments (Table 2).

### 3.4. Population Parameters

The control group showed a higher intrinsic rate of increase (*r*) of 0.20/day, whereas the LC_50_-treated individuals had a lower rate of 0.18 (Table 3). The lambda (*λ*) values indicated that the finite rate of increase (per day) was not significantly affected. Individuals treated with LC_50_ had a net reproductive rate (*R*_0_) of 13.22–15.42 offspring per day, significantly lower than the control group’s 27.30–29.32 offspring per day. There was no statistically significant difference between the two groups regarding mean generation times. The treated groups had a considerably longer mean generation time than the LC_50_-treated groups (*T* = 16.47–16.58 days) (Table 3).

### 3.5. Age-Stage Specific Maternity (lxmx)

**Figure 2:**
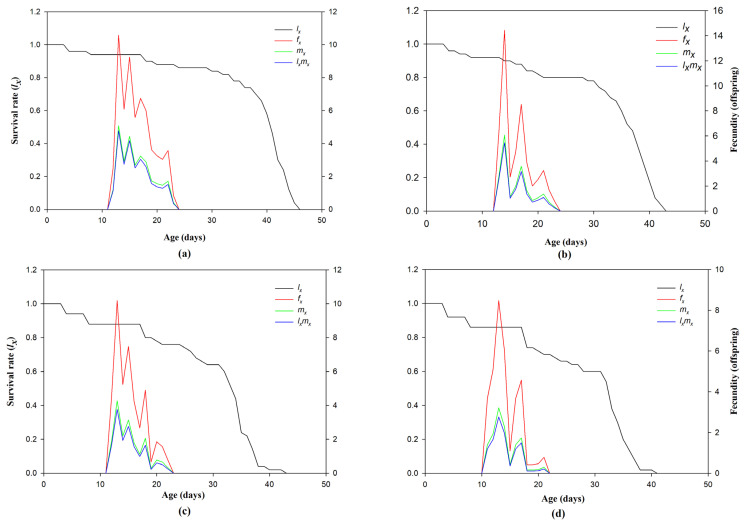
Survival rate (*lx*), maternity rate (*mx*), and their product (*lxmx*) in the F_1_ progeny of chlorantraniliprole-treated larvae of *Ae. aegypti*. Control (**a**), LC_10_ (**b**), LC_30_ (**c**), and LC_50_ (**d**).

**Figure 3:**
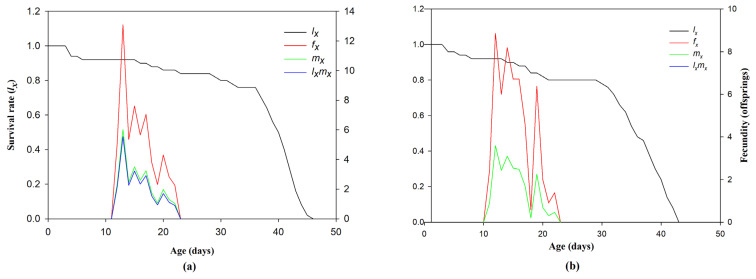

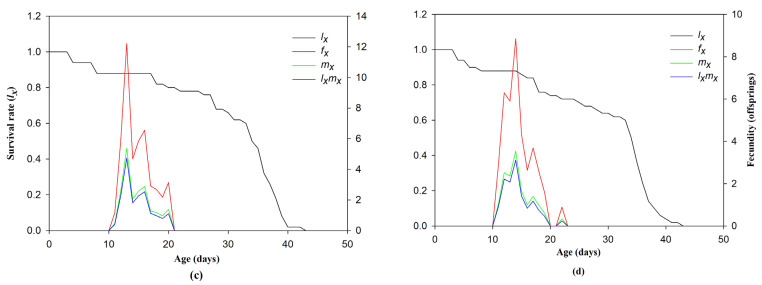
Survival rate (*l_x_*), maternity rate (*m_x_*) and their product (*l_x_m_x_*) in the F_2_ progeny of chlorantraniliprole-treated larvae of *Ae. aegypti*. Control (**a**), LC_10_ (**b**), LC_30_ (**c**), and LC_50_ (**d**).

A fecundity (*fx*) peak of 10.58/day was registered for 13-day-old females of the control group. A decline became apparent between days 16 and 24. Over time, the age-specific survival rate (*lx*) of the control group declined, reaching approximately 45 days. The maximum age-specific fertility (*mx*) score was 5.08 on day 13 of the control treatment. The age-specific maternity rate (*lxmx*) drastically decreased in the LC_50_ treated individuals, from 4.78 offspring per day in the control group to 2.76 offspring per day (Figure 2 and Figure 3).

### 3.6. Effect of Chlorantraniliprole Exposure on Enzyme Activity

**Figure 4:**
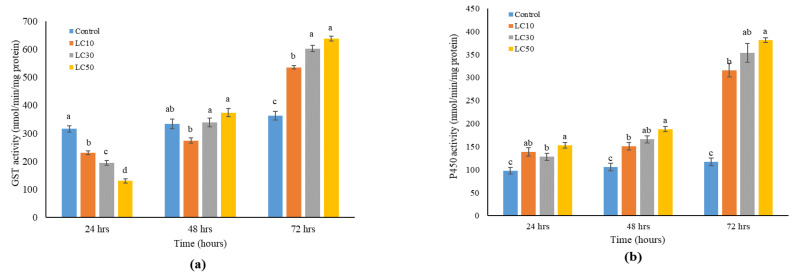
(**a**) GST and (**b**) P450 activity in *Ae. aegypti* larvae treated with chlorantraniliprole. Each value represents the mean (±SE) of five replications. Different letters over the bars indicate a significant difference at *p* < 0.05 (Tukey’s HSD).

The enzymatic activity of *Ae. aegypti* detoxification metabolism enzymes (GST and P450) were enhanced following exposure to chlorantraniliprole at sublethal concentrations (Figure 4). The study revealed that the action of chlorantraniliprole is influenced by time, as evidenced by the correlation between GST activity and the duration of exposure (*p* < 0.05). Based on the findings, chlorantraniliprole exhibited a swift impact on reducing GST activities in the tested larvae within 24 h, specifically in the range of 130–230 nmol/min/mg protein. After 24 h of treatment, the GST activity in the control group increased to 316 nmol/min/mg protein. Significant increases in GST activity were detected in the treated larvae at 48 and 72 h (*p* < 0.05). The LC_50_ treatment had the highest level of activity.

Similarly, chlorantraniliprole showed an immediate impact on enhancing P450 activities in the test larvae within 24 h, ranging from 128 to 153 nmol/min/mg protein. After 24 h of treatment, mosquitoes of the control group exhibited reduced P450 activity, measuring 98 nmol/min/mg protein. A significant rise in P450 activity was detected in the treated larvae after 48 and 72 h (*p* < 0.05). The LC_50_ treatment exhibited the highest activity level, measuring at 381 nmol/min/mg protein.

## 4. Discussion

Using highly effective insecticides with minimal impact on non-target organisms is critical for effectively managing harmful insect populations [37]. The data indicated that the application of chlorantraniliprole had a substantial impact on decreasing the duration of the larval stage of mosquitoes (>5.00%). In addition, there was a significant decrease (>30.00%) in the oviposition period. The duration of the larvae was found to be considerably decreased in our study of sublethal treatments. Previous studies as conducted by Lutz et al. [38] found similar results, which suggested that giving chlorantraniliprole to *Plutella xylostella* L. at doses in sublethal doses resulted in a decrease in the oviposition period (about 50.00%). In previous studies conducted by Guo, chlorantraniliprole showed increased larval duration. The discrepancy may be due to the more susceptible generation used in our study (>15 generation). A more susceptible generation can have a more severe effect on the target insect population [39]. In the offspring of insects exposed to low doses of chlorantraniliprole, the decrease in male longevity was more than 20%, while the decrease in female longevity was more than 16%, and the number of eggs produced per female decreased by more than 40%. The results align with the studies conducted by Nawaz et al. [40] and Zhang et al. [41], which demonstrated a decline in the fecundity of exposed insects (40–50%) whose parents were subjected to chlorantraniliprole. Chlorantraniliprole exhibits persistent efficacy against many pests, such as the oblique banded leafroller, the grapevine moth, and white grubs [42]. Insects use significant amounts of energy to detoxify harmful chemicals from their bodies. This process has a negative impact on their growth and development, and there is a compromise in how they allocate their energy resources [43].

The net reproductive rate (*R*_0_) (>50%) and the mean generation time (>11%) were considerably reduced in the offspring of mosquitoes exposed to sublethal doses of chlorantraniliprole. Han et al. [44] found that important biological factors such as *R*_0_, *T*, and fecundity were drastically reduced when diamondback moths were exposed to a sublethal dose of chlorantraniliprole. Likewise, the *l_x_m_x_* was reduced in the insects that received sublethal amounts of chlorantraniliprole. Wang et al. [45] showed that treating *P. xylostella* with low doses of chlorantraniliprole resulted in suppressed feeding, reduced egg hatching, and increased mortality. Estimating the population characteristics of insects can provide insights into their relative fitness, which in turn aids in predicting their environmental adaptability, proliferation, and decline [34,46].

When pests take insecticides, they undergo physiological changes to detoxify and remove the toxins [47,48]. The identification of the origin of pesticide resistance can be aided by evaluating detoxifying enzymes. These enzymes convert and break down pesticide compounds, reducing their harmful effects or protecting insect target areas by preventing their action [49]. After 24 h, GST activity in mosquitoes of all three treated groups decreased compared to activity in mosquitoes of the control group, but afterwards GST activity increased. Similar GST activity has also been reported by Gui et al. [50] in silkworms. Initially decreased and then increased activity can be attributed to initial detoxification overload, cellular stress response, and adaptive response [51,52]. A significantly increased activity of the P450 enzyme was observed immediately after 24 h of treatment in treated groups compared to the control. The results are in agreement with the previous study conducted by Cao et al. [53] and Haas et al. [54], where an increased level of P450 was found after the application of chlorantraniliprole. The findings indicated that P450 had a role in the detoxification of chlorogenic acid, as reported by Wang et al. [55]. Exposure to stress caused by sublethal concentrations can diminish the capacity of the detoxifying metabolism. The detoxification capacity is typically associated with heightened resistance to insecticides and instances of failure in field management [56].

Using *Ae. aegypti* as a model organism, this study provides thorough observations on the toxicity and sublethal effects of chlorantraniliprole, thereby laying a strong basis for understanding its consequences in mosquito control. This paradigm also fits other mosquito species. While controlled conditions yield precise results, later studies can investigate field-based studies to grasp long-term dynamics and more general ecological consequences. Moreover, investigating interactions with other control techniques and environmental factors could help to better incorporate them into integrated pest management plans, thereby strengthening the basis for more effective and sustainable mosquito control.

## 5. Conclusions

The study demonstrated that sublethal doses of chlorantraniliprole significantly affected the development time and fecundity of the F1 generation of *Ae. aegypti* mosquitoes. Exposure to chlorantraniliprole increased the activity of detoxification metabolism enzymes: specifically, GST and P450 activities rose following treatment with sublethal amounts of the drug. Chlorantraniliprole has demonstrated long-term residual efficacy against *Ae. aegypti* mosquitoes.

## Figures and Tables

**Table 1:** Toxicity of chlorantraniliprole against *Ae. aegypti.*

Insecticide	N	LC_10_ (95% FL) (mg L^−1^)	LC_30_ (95% FL) (mg L^−1^)	LC_50_ (95% FL) (mg L^−1^)	*X* ^2^	Df	Slope ± SE	*p*-Value
Chlorantraniliprole	360	0.202 (0.145–0.257)	0.373 (0.300–0.443)	0.569 (0.483–0.663)	3.58	16	2.85 ± 0.29	0.22

N = total number.

**Table 2:** Fecundity and duration of various life-history parameters (mean ± SE) of the progeny of chlorantraniliprole-treated populations of *Ae. aegypti*.

Parameters	Progeny	Treatments			
		Control	LC_10_	LC_30_	LC_50_
Egg (days)	F_1_	2.32 ± 0.25 aA	2.39 ± 0.28 aA	2.46 ± 0.33 aA	2.64 ± 0.38 aA
	F_2_	2.19 ± 0.25 aA	2.21 ± 0.30 aA	2.40 ± 0.32 aA	2.52 ± 0.35 aA
Larva (days)	F_1_	6.61 ± 0.07 aA	6.33 ± 0.08 bA	6.38 ± 0.07 bA	6.25 ± 0.09 bA
	F_2_	6.55 ± 0.07 aA	6.14 ± 0.08 bB	6.42 ± 0.07 aA	6.21 ± 0.05 bA
Pupa (days)	F_1_	2.71 ± 0.06 aA	2.42 ± 0.07 aB	2.53 ± 0.06 aA	2.44 ± 0.08 aA
	F_2_	2.48 ± 0.08 aA	2.68 ± 0.07 aA	2.43 ± 0.06 aA	2.36 ± 0.07 aA
Male longevity (days)	F_1_	40.95 ± 1.10 aA	37.25 ± 1.08 bA	32.83 ± 1.08 bA	32.58 ± 1.06 cA
	F_2_	40.20 ± 1.15 aA	37.45 ± 1.13 aA	34.38 ± 1.01 bA	33.88 ± 1.17 bA
Female longevity (days)	F_1_	40.33 ± 0.82 aA	36.18 ± 1.30 bA	34.80 ± 0.87 bA	33.73 ± 0.98 bA
	F_2_	39.82 ± 0.89 aA	36.09 ± 1.33 bA	35.81 ± 0.81 bA	34.20 ± 1.36 bA
Oviposition days	F_1_	2.69 ± 0.11 aA	2.58 ± 0.13 aA	2.25 ± 0.16 bA	1.84 ± 0.14 cA
	F_2_	2.65 ± 0.11 aA	2.56 ± 0.13 bA	2.23 ± 0.15 bA	1.90 ± 0.13 cA
APOP (days)	F_1_	2.73 ± 0.09 aA	2.85 ± 0.11 aA	2.70 ± 0.08 aA	2.61 ± 0.06 aA
	F_2_	2.71 ± 0.06 aA	2.81 ± 0.11 aA	2.59 ± 0.08 aA	2.57 ± 0.13 aA
TPOP (days)	F_1_	12.66 ± 0.07 aA	13.78 ± 0.09 aA	13.30 ± 0.13 aA	13.90 ± 0.05 aA
	F_2_	12.90 ± 0.07 aA	13.27 ± 0.08 aA	13.01 ± 0.05 aB	12.95 ± 0.07 aB
Fecundity (eggs/ female)	F_1_	61.08 ± 3.98 aA	48.81 ± 3.74 bA	44.04 ± 3.80 bA	34.78 ± 3.24 cA
	F_2_	59.34 ± 4.02 aA	52.81 ± 3.96 aA	47.18 ± 4.02 bA	38.55 ± 3.32 bA

APOP: Adult pre-oviposition period of a female adult; TPOP: Total pre-oviposition period of female counted from birth. Means in the same row followed by different small letters or means in the column followed by different capital letters are significantly different (*p* < 0.05) using the bootstrap test.

**Table 3:** Influence of chlorantraniliprole on demographic parameters (mean ± SE) of the progeny of *Ae. aegypti*.

**Parameters**	Progeny	**Treatments**			
		Control	LC_10_	LC_30_	LC_50_
*r*	F_1_	0.20 ± 0.01 aA	0.18 ± 0.02 aA	0.18 ± 0.02 aA	0.17 ± 0.01 aA
	F_2_	0.20 ± 0.01 aA	0.20 ± 0.01 aA	0.19 ± 0.01 aA	0.18 ± 0.02 aA
*λ*	F_1_	1.22 ± 0.02 aA	1.20 ± 0.01 aA	1.20 ± 0.01 aA	1.19 ± 0.02 aA
	F_2_	1.22 ± 0.01 aA	1.22 ± 0.01 aA	1.21 ± 0.01 aA	1.20 ± 0.02 aA
*R*_0_	F_1_	29.32 ± 4.69 aA	21.48 ± 3.79 bA	18.50 ± 3.46 bA	13.22 ± 2.67 cA
	F_2_	27.30 ± 4.57 aA	23.24 ± 4.09 bA	20.76 ± 3.73 bA	15.42 ± 2.97 cA
*T*	F_1_	16.58 ± 0.27 aA	16.63 ± 0.21 aA	15.73 ± 0.29 bA	14.70 ± 0.33 cA
	F_2_	16.47 ± 0.24 aA	16.05 ± 0.27 aB	15.34 ± 0.28 bA	14.98 ± 0.33 bA

*r* = The intrinsic rate of increase (per day). *λ* = The finite rate of increase (per day). *R*_0_ = The net reproductive rate (offspring/individual). *T* = The mean generation time (days). Using the bootstrap, means in the same row followed by different small letters or means in the column followed by different capital letters are statistically different (*p* < 0.05).

## Data Availability

The data that support the findings of this study are available from the corresponding authors upon reasonable request.
